# The effects of mutant Ras proteins on the cell signalome

**DOI:** 10.1007/s10555-020-09912-8

**Published:** 2020-07-09

**Authors:** Tamás Takács, Gyöngyi Kudlik, Anita Kurilla, Bálint Szeder, László Buday, Virag Vas

**Affiliations:** 1 Institute of Enzymology, Research Centre for Natural Sciences, Budapest, Hungary; 2 Department of Medical Chemistry, Semmelweis University Medical School, Budapest, Hungary

**Keywords:** Mutant Ras protein, Signal transduction, Phosphorylation, Tumorigenesis

## Abstract

The genetic alterations in cancer cells are tightly linked to signaling pathway dysregulation. Ras is a key molecule that controls several tumorigenesis-related processes, and mutations in RAS genes often lead to unbiased intensification of signaling networks that fuel cancer progression. In this article, we review recent studies that describe mutant Ras-regulated signaling routes and their cross-talk. In addition to the two main Ras-driven signaling pathways, i.e., the RAF/MEK/ERK and PI3K/AKT/mTOR pathways, we have also collected emerging data showing the importance of Ras in other signaling pathways, including the RAC/PAK, RalGDS/Ral, and PKC/PLC signaling pathways. Moreover, microRNA-regulated Ras-associated signaling pathways are also discussed to highlight the importance of Ras regulation in cancer. Finally, emerging data show that the signal alterations in specific cell types, such as cancer stem cells, could promote cancer development. Therefore, we also cover the up-to-date findings related to Ras-regulated signal transduction in cancer stem cells.

## Introduction

**Fig. 1:**
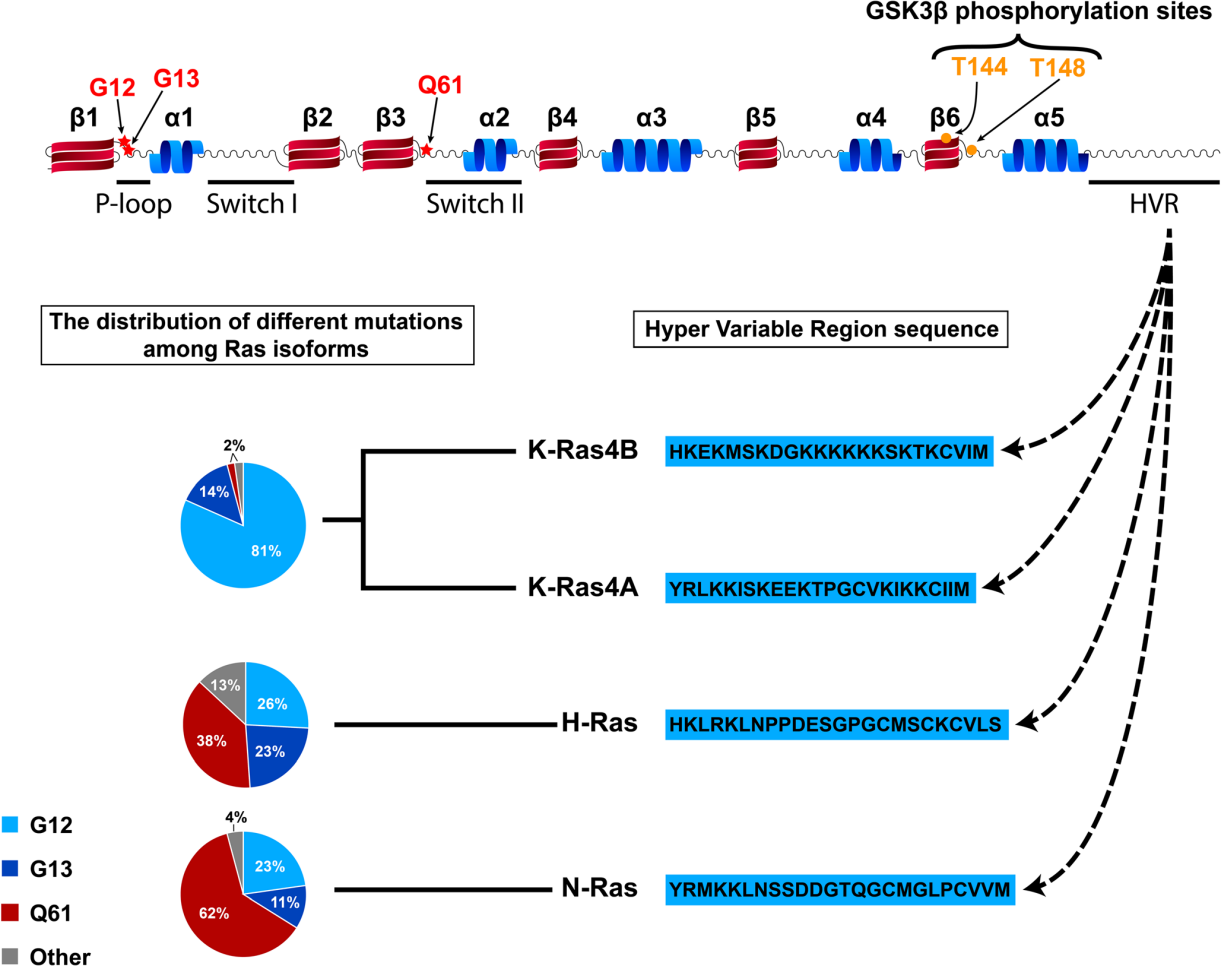
Schematic representation of Ras proteins. The GTPase domain consists of six β-strands (red labeled) and five α-helices (blue labeled). Three inter -β-strand and α-helix loops are highlighted, i.e., the P-loop, the switch I, and the switch II regions. The P-loop binds the beta phosphate of guanosine phosphates. The switch I and switch II regions undergo conformational changes during GTP-GDP hydrolysis and determine the interactions of Ras partner proteins. The positions of the three most frequent codon mutations are labeled with red letters, i.e., the glycines at codons 12 and 13 and the glutamine at codon 61. The two threonines at positions 144 and 148 (labeled with orange letters) can be phosphorylated by GSK3β kinase and organize the ubiquitination of Ras to regulate degradation. Each Ras protein has an isoform-specific hypervariable region (HVR) at the C-terminus that can be post-translationally modified through palmitoylation, farnesylation, acetylation, methylation, or prenylation. The sequences of the four HVR regions of K-Ras4B, K-Ras4A, H-Ras, and N-Ras (depicted in blue) determine the PM and lipid raft localization of Ras. The pie charts show the G12, G13, and Q61 mutation frequencies in the given isoforms

Since the discovery of Ras as a key regulator of retrovirus-induced cell proliferation, a massive scientific effort has been devoted to identifying its critical roles in biology, especially in oncogenesis. Ras proteins are specialized guanine nucleotide-binding and hydrolyzing molecules that belong to the small G-protein superfamily [1]. The human genome contains three highly related RAS genes, namely, KRAS (Kirsten rat sarcoma viral oncogene homolog), NRAS (neuroblastoma RAS viral oncogene homolog), and HRAS (Harvey rat sarcoma viral oncogene homolog), which encode four highly homologous proteins, namely, H-Ras, K-Ras4A and K-Ras4B (two splice variants of K-Ras), and N-Ras. Ras proteins have a molecular weight of approximately 21 kDa, and each contains a soluble catalytic G-domain and a C-terminal flexible hypervariable region (HVR) (Fig. 1.)

The majority of the mutations in RAS genes that lead to oncogenic events are single-base substitutions in well-described positions in the G-domain. The most frequent oncogenic mutational hotspots are at codons 12, 13, and 61 in all four Ras isoforms. Less frequently, other codons in RAS genes are also affected by mutations, as summarized in Table 1. Oncogenic mutations in the G-domain allow Ras protein to constitutively activate downstream effectors. Interestingly, the different amino acid substitutions in the mutational hotspots distinctly alter the GTPase function of Ras and its interactions with signaling molecules. Moreover, mutant Ras proteins differently activate the RAF/MEK/ERK kinase cascade and other non-canonical downstream signaling molecules. The interdependence and cross-talk of downstream effectors regulated by mutant Ras proteins and recent advances in understanding the broad spectrum of the effects of Ras in cell biology are discussed here.

**Table 1:** An overview of the signaling effects of the most highly studied RAS mutations

RAS isoforms and chromosome location	Codon	Amino acid substitution	Signaling alterations
KRAS Chromosome 12 Short arm, position 12.1	12: GGT (glycine)	Alanine (G12A)	No significant association with ERK activation [2]
		Cysteine (G12C)	Elevated MAPK signaling [3]
		Aspartic acid (G12D)	Increased GTP-bound state [4]; strong association with ERK activation [2]; constitutively active MAPK signaling [5]
		Arginine (G12R)	Decreased MAPK signaling [3]
		Serine (G12S)	Decreased MAPK signaling [3]; oncogenic effects through overactive MAPK signaling and ELK1 activity [6]
		Valine (G12V)	Increased GTP-bound state [7]; no significant association with ERK activation [2]; constitutively active MAPK signaling [5]
	13: GGC (glycine)	Cysteine (G13C)	Increased GTP-bound state [7]
		Aspartic acid (G13D)	Fast GDP/GTP exchange [8]; constitutively active MAPK signaling [5]
	61: CAA (glutamine)	Histidine (Q61H)	No significant association with ERK activation [2]
		Leucine (Q61L)	Elevated ERK phosphorylation, low level of GAP-mediated hydrolysis [8]
	14: GTA (valine)	Isoleucine (V14I)	Increased GTP-bound state [9]
	18: GCC (alanine)	Aspartic acid (A18D)	No significant change in GTP-bound state [7]
	19: TTG (leucine)	Phenylalanine (L19F)	Increased GTP-bound state [10]
	22: CAG (glutamine)	Lysine (Q22K)	Increased GTP-bound state [4]
	59: GCA (alanine)	Threonine (A59T)	Oncogenic effects through overactive MAPK signaling and ELK1 activity [6]
	117: AAA (lysine)	Asparagine (K117N)	Moderate increase in GTP-bound state, elevated ERK phosphorylation [4]
	146: GCA (alanine)	Threonine (A146T)	Moderate increase in GTP-bound state, elevated ERK phosphorylation [4]
HRAS Chromosome 11 Short arm, position 5	12: GGC (glycine)	Alanine (G12A)	Increased GTP-bound state and no change in MAPK signaling, enhanced PI3K signaling [11] Increased GTP-bound state, enhanced ERK and c-Jun N-terminal kinase activity [12]
		Cysteine (G12C)	Increased GTP-bound state, enhanced ERK and c-Jun N-terminal kinase activity [12]
		Aspartic acid (G12D)	Increased GTP-bound state, enhanced ERK and c-Jun N-terminal kinase activity [12]
		Arginine (G12R)	Upregulation of the MKP3 gene via the activation of the PI3K-AKT pathway causing impaired FGF2-induced ERK1/2 phosphorylation [13]
		Serine (G12S)	Increased GTP-bound state, enhanced MAPK signaling, and strong phosphorylation of AKT in COS-7 cells, while no change in MAPK signaling and enhanced PI3K signaling in patient-derived cells [11]; increased GTP-bound state, enhanced ERK and c-Jun N-terminal kinase activity [12]
		Valine (G12V)	Constitutively active MAPK signaling [5]; enhanced MAPK signaling [14]; increased GTP-bound state, enhanced MAPK signaling, and strong phosphorylation of AKT in mutant COS-7 cells, while no change in MAPK signaling and enhanced PI3K signaling in patient-derived cells [11]; increased GTP-bound state, enhanced ERK and c-Jun N-terminal kinase activity [12]
	13: GGT (glycine)	Cysteine (G13C)	Increased GTP-bound state, no change in MAPK signaling, enhanced PI3K signaling [11]; increased GTP-bound state, enhanced ERK and c-Jun N-terminal kinase activity [12]
		Aspartic acid (G13D)	Increased GTP-bound state, no change in MAPK signaling, enhanced PI3K signaling [11]; increased GTP-bound state, enhanced ERK and c-Jun N-terminal kinase activity [12]
		Arginine (G13R)	Constitutive activation of MAPK signaling [15]; constitutive activation of MAPK signaling [16]
	61: CAG (glutamine)	Lysine (Q61K)	Enhanced PI3KAKT-mTOR and MAPK signaling [17]; constitutive activation of MAPK signaling [16]
		Leucine (Q61L)	Enhanced MAPK signaling [18]
		Arginine (Q61R)	Increased GTP-bound state, enhanced PI3KAKT-mTOR and MAPK signaling [17, 19]; constitutive activation of MAPK signaling [16]
NRAS Chromosome Short arm, position 13.2	12: GGT (glycine)	Aspartic acid (G12D)	Activation of MAPK signaling [20] Oncogenic effects independent from MAPK signaling and ELK1 activity [6]
		Serine (G12S)	Effects on NRAS function have not been elucidated [21]
		Valine (G12V)	Induction of PI3K/AKT/rS6 signaling [22]; enhanced MAPK signaling [14];increased GTP-bound state, upregulated MAPK signaling and AKT phosphorylation [23]
	13: GGT (glycine)	Aspartic acid (G13D)	Induction of MAPK signaling [24]; no significant association with ERK activation [2]
		Arginine (G13R)	No significant association with ERK activation [2]
	61: CAA (glutamine)	Histidine (Q61H)	MEK-independent regulation of ERK: phosphorylation of ERK1/2 without phosphorylation of MEK1/2 [25]; no significant association with ERK activation [2]
		Lysine (Q61K)	Enhanced MAPK signaling [26]; no significant association with ERK activation [2]
		Leucine (Q61L)	Enhanced MAPK signaling [22]
		Proline (Q61P)	Increased GTP-bound state [27]
		Arginine (Q61R)	Activation of MAPK and PI3K-AKT-mTOR signaling [28]; no significant association with ERK activation [2]
	60: GGA (glycine)	Glutamic acid (G60E)	Increased GTP-bound state [29]; increased GTP-bound state, upregulated MAPK signaling, and no effect on AKT phosphorylation [23]
	146: GCC (alanine)	Threonine (A146T)	Increased levels of activated Ras, hyperactive MAPK signaling, enhanced PI3K signaling [30]

The data presented here sometimes appear contradictory, possibly due to differences in the model systems used, e.g., expression of the mutant gene (ectopic vs. endogenous, transient vs. sustained), the use of cell lines or patient-derived cells, different culture conditions (medium with or without serum, use of different growth factors), or the cell context (transformed vs. untransformed environments) in which the mutations exert their effects. All of these factors can contribute to the signaling alterations observed for each mutation present in Ras proteins [14, 23, 31].

## Structural basis of wild-type and mutant Ras activation kinetics

Several studies have demonstrated that the Ras protein level is elevated in cancer tissues and that increased Ras expression is correlated with poor prognosis [32, 33]. As the mechanisms regulating Ras protein level have significant pathological implications, the factors and signaling pathways influencing the stable and unstable degradable forms of Ras are possible determinants of cancer progression. Ras stability depends on the phosphorylation status of the protein on two threonine residues at positions 144 and 148 (Fig. 1). Once Ras is phosphorylated by GSK3β kinase at these positions, phospho-Ras can bind the β-TrCP protein [34]. An E3 ligase recognizes the β-TrCP-phospho-Ras complex and ubiquitinates Ras, thus leading to Ras degradation by the proteasome. [33] The regulation of GSK3β kinase depends on Wnt/β-catenin signaling to regulate the ubiquitination-dependent degradation of both the wild-type and mutant forms of Ras [35]. Although phosphorylation-dependent degradation presumably has a role in the regulation of mutant and wild-type Ras protein levels, interestingly, the mutational hotspots leading to Ras-mediated cancer development do not affect the GSK3β-targeted threonines in Ras. Therefore, it is highly probable that the activation level of accessible Ras protein is the major decisive factor in oncogenesis rather than the stability of Ras.

**Fig. 2:**
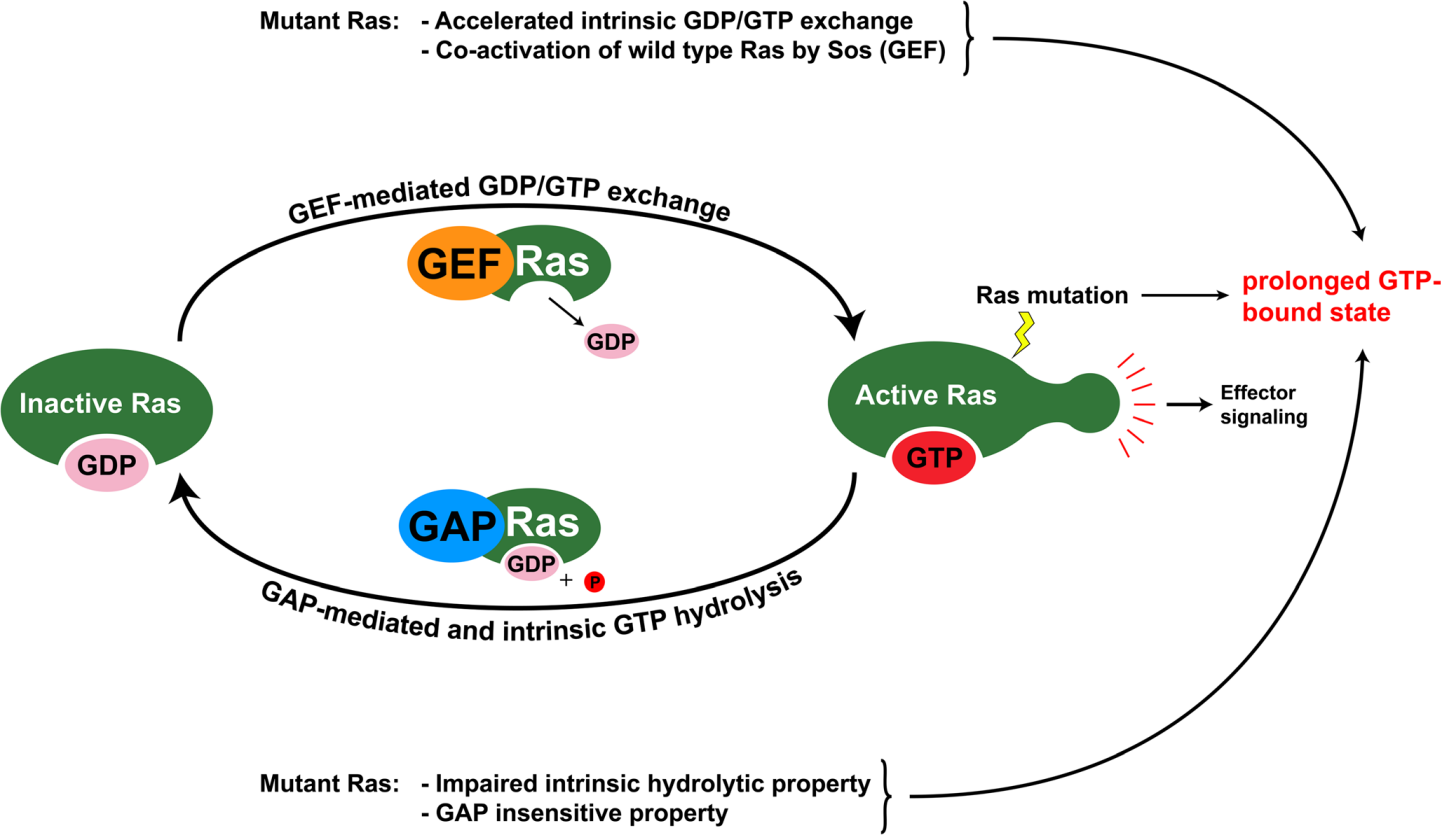
Schematic representation of the mechanism leading to constitutively active forms of mutant Ras. The transition from inactive GDP-bound to active GTP-bound Ras is regulated by several factors, including GEFs and GAPs. Oncogenic mutant Ras proteins could remain in a prolonged active form. Mutations in Ras can result in an accelerated intrinsic GDP/GTP exchange rate or impairment of its intrinsic hydrolytic activity. In addition to changing the intrinsic enzymatic activity of Ras, oncogenic Ras mutations can also alter its sensitivity to GAP and GEF activity

The active state of wild-type Ras depends on its intrinsic ability to bind and hydrolyze GTP via the conserved 20-kDa G-domain. The Ras intrinsic GTPase activity is accelerated by the GEF/GAP system (guanine nucleotide exchange factors and GTPase-activating proteins, respectively), which fuel the cycling between inactive GDP-Ras and active GTP-Ras. GEFs are the main activators of Ras via catalyzation of GDP release and GTP loading in the GTPase domain. Ras can bind GTP with high affinity, and GEFs can sterically displace the catalytic magnesium ion from the G-domain and restructure the nucleotide binding site, thus leading to the exchange of GDP to GTP. To downregulate Ras signaling, GAP proteins accelerate Ras-dependent GTP hydrolysis. A common feature of oncogenic Ras mutations is that they affect the nucleotide-binding site of Ras and lead to aberrant constitutive activation. The most common mutations, which are in codons 12, 13, and 61, lie in the G-loops of the protein by which Ras interacts with GDP and GTP. Although it is widely accepted that most Ras mutations stabilize the protein in its active state and prolong its downstream signaling, less is known about the mechanisms by which each unique nucleotide substitution can influence Ras signalome activation. Here we assembled the current evidence regarding how specific single-nucleotide substitutions in mutant Ras help to maintain the protein in its active state. It is already known that point mutations can directly prolong the GTP-bound state of Ras as reported in two independent studies comparing the features of different Ras mutants. Stolze et al. reported (based on the work in MCF10A cells) that the G13C, G12C, and G12V substitutions, along with two rare substitutions (Q61H and K117N), lead to higher levels of GTP binding relative to that of wild-type Ras [7]. Additionally, Janakiraman et al. found that when the G12D and Q22K mutations were present in HEK-293T cells, Ras was more robustly preserved in a GTP-bound state [4]. One mechanism behind how mutant Ras remains constitutively activated is via a reduction in its intrinsic GTPase activity, as reported by Hunter et al. for single-nucleotide substitutions in codon 12. They showed that most Ras proteins carrying different mutations at codon 12 have dramatically decreased rates of intrinsic GTP hydrolysis [8, 36]. Furthermore, characterization of another frequently mutated codon at position 61 also revealed decreased intrinsic GTPase activity [37, 38]. Consistent with this concept, in addition to effects on the intrinsic activity, several Ras mutants have slower GAP-mediated GTP hydrolysis rates [8]. The significant GAP-insensitive property of mutant Ras, combined with its impaired intrinsic GTPase activity, allows it to remain longer in the GTP-bound active state (Fig. 2). It was also recently shown that mutant Ras can increase the level of activated wild-type Ras [39]. In line with this concept, Jeng et al. found that G12V mutant KRas upregulates wild type H-Ras and N-Ras activation level. They also showed that SOS (a GEF for Ras) can mediate such cross-activation by serving as a binding platform between mutant and wild-type Ras [39]. Last but not least, a faster GDP to GTP exchange rate is also a possible mechanism allowing constitutive activation of mutant Ras. For example, it has been demonstrated that the intrinsic GEF-independent GDP exchange rate in G13D K-Ras mutant is an order of magnitude higher than that of wild-type Ras [8, 40].

## Specialties of mutant Ras-driven signaling

**Fig. 3:**
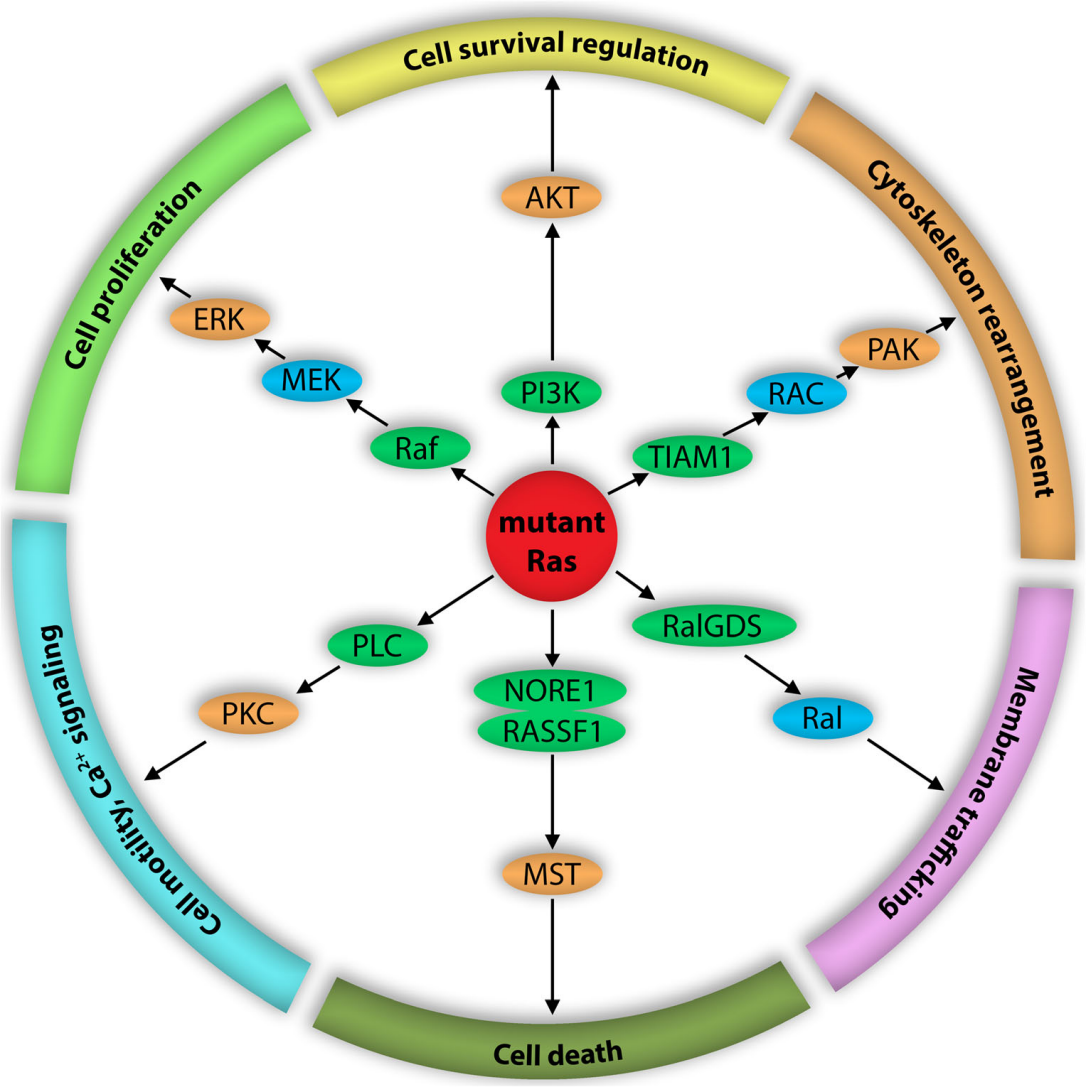
Signaling networks involved in Ras-driven oncogenesis. This figure summarizes the core members of the signaling pathways radiating from mutant Ras. The two robust Ras-driven signaling routes are the RAF/MEK/ERK and PI3K/AKT pathways, which regulate diverse cellular processes, particularly cell proliferation and cell survival regulation, respectively. Other Ras activation-dependent signaling routes are less studied. The TIAM1/RAC/PAK pathway primarily controls cytoskeleton rearrangement in certain cells, and the RalGDS/Ral pathway mostly influences membrane trafficking. The NORE1/RASSF1/MST signaling pathway is a regulator of cell death processes. Mutant Ras can also mediate signaling via PLC/PKC molecules to influence Ca+-dependent signaling in cancer cells

In the presence of growth factors (GFs), several intracellular signaling pathways are initiated in normal cells. In the first step, GF binding converts the receptor into an active state via autophosphorylation and dimerization, leading to recruitment of adaptor proteins such as Grb2 and Shc to the dimer’s cytoplasmic tail. Via Grb2, GEF proteins (e.g., SOS) can localize to the membrane and facilitate Ras activation. When wild-type Ras is in its GTP-bound state, it can assemble different signaling molecules at the membrane. The various Ras-associated effector proteins then promote canonical and non-canonical downstream Ras signaling (Fig. 3).

## The MAPK pathway

When Ras is activated by either GEFs or mutations, GTP-bound Ras undergoes conformational changes in the switch regions that allow RAF binding. RAF activation requires dimerization of GTP-bound Ras molecules. In the case of constitutively active mutant Ras and its dimers, the size of the membrane nanoclusters increases and more RAF molecules can be recruited. Therefore, under Ras mutant oncogenic conditions, Ras-dependent RAF signal transduction can be robustly amplified. RAF is a serine/threonine kinase with three paralogs, i.e., A-RAF, B-RAF, and C-RAF. Among them, A-RAF has the lowest and B-RAF has the highest kinase activity [41]. Notably, like Ras, RAF proteins are also oncogenes, and B-RAF is the most frequent mutant RAF variant in cancers [42]. Mutant RAF proteins either bypass the Ras-initiated RAF dimerization step for their activation or have increased kinase activity, and oncogenic RAF mutations can lead to enhanced signaling via both mechanisms [43]. Dimerized GTP-Ras allows RAF to be released from its autoinhibited state. RAF kinase domains activate mitogen-activated and extracellular signal-regulated kinases (MEK) by phosphorylating two conserved serine residues in the catalytic domain of MEK. At this step in the MAPK pathway, the MEK subtypes MEK1 and MEK2 dual-specificity kinases can catalyze the phosphorylation of ERK1 and ERK2 on threonine and tyrosine residues. The next step in MAPK signaling involves a branching in the signal propagation pathway after which ERK mediates the phosphorylation of multiple cytoskeletal, cytoplasmic, and nuclear partner molecules. The most studied molecular targets of ERK kinase activity are nuclear transcription factors. Upon ERK phosphorylation, ERK forms dimers and translocates to the nuclear membrane, leading to modulation of c-Myc, c-Fos, c-Jun, and Elk-1 activities. When these transcription factors are activated and bind to the promoters of their target genes, the expression of well-known cell survival and cell cycle regulators is induced. Additionally, cytoplasmic ERK can modulate other signaling molecules, including cytoskeletal microtubule-associated proteins (MAPs), ribosomal s6 kinase (RSK), mitogen- and stress-activated protein kinases (MSKs), and cytosolic phospholipase A2 (Cpla2). ERK activation also represents a feedback regulatory loop since ERK can hyperphosphorylate signaling molecules upstream in the MAPK pathway. This process is critical in the deregulation of the Ras-initiated signaling as the hyperphosphorylation of GEFs (i.e., SOS1) disrupts their association with Ras and Ras localization at the membrane [44]. More than 250 ERK target proteins have already been identified, highlighting the robustness of the effects of ERK during MAPK signaling (reviewed by [45]).

The ultimate outcome of the wild-type Ras-driven MAPK pathway is context dependent and can also lead to the initiation of diverse genetic programs associated with cell growth, cell migration, cell cycle, and cell survival. Moreover, Ras and its downstream MAPK effectors were also connected to the regulation of circadian rhythm [46]. Since mutant Ras proteins are in a constitutively active, GTP-bound state, one explanation for their oncogenicity is that they initiate increased MAPK signaling [47]. Although this presumption is highly likely to be valid, not all Ras mutants accelerate the MAPK pathway. For instance, Burd et al. showed that not all mutant NRAS proteins (i.e., those with mutations at codons 12, 13, and 61) can induce increased ERK phosphorylation [48]. A study that analyzed the affinity of mutant Ras proteins for RAF demonstrated that KRAS codon 12 mutants do not exhibit a very high affinity for RAF even though their GTPase activity is low [8]. Riquelme et al. also confirmed that not all oncogenic K-Ras mutants activate and enhance MAPK signaling by showing that the level of phospho-MEK1/2 is increased in NSCLC cells harboring the KRAS G12C and KRAS G12D mutations but decreased in cells harboring the KRAS G12R and G12S [3]. By contrast, a recent study using NIH3T3 cells showed that KRAS G12S-expressing cells had elevated p-ERK levels, suggesting that this mutant in this setting exerts its oncogenic phenotype via MAPK signaling [6].

Taken together, the data summarized here show that single amino acid substitutions at codons 12, 13, or 61 in RAS can stabilize the active GTP-bound state of RAS protein but that not all mutations result in elevated MAPK signaling (Table 1); therefore, it is clear that this type of signaling is highly context dependent and is influenced by the level of mutant Ras protein or by the given cell type.

## The PI3K/AKT/mTOR signaling pathway

The other major signaling pathway initiated by Ras is the PI3Kα/AKT/mTOR pathway, and among many other cellular processes, this pathway plays crucial roles in cell survival and apoptosis inhibition [49].

It has been demonstrated that Ras isoforms differentially activate the two canonical Ras signaling cascades and that there are also differences between the specific Ras mutants in their signal initiation. According to the model proposed by Nussinov et al., the K-Ras4B isoform might be the most potent activator of the PI3Kα/AKT pathway [50]. Based on the observations that K-Ras4B can form a multimeric complex with CaM and PI3Kα and that this mode of PI3K activation is independent of the RTK signal, the authors proposed that K-Ras most efficiently triggers the PI3Kα/AKT pathway [51].

The PI3Kα protein is a lipid kinase consisting of catalytic and regulatory subunits. It converts phosphatidylinositol (4,5)-bisphosphate (PIP2) to phosphatidylinositol (3,4,5)-trisphosphate (PIP3) at the cell membrane. PI3Kα can directly bind Ras via the Ras-binding domain (RBD) in its p110 catalytic subunit, and this interaction leads to PI3Kα activation. The mechanism of this activation remains unclear; however, it has been established that PI3Kα recruitment to the cell membrane by Ras has a key role since Ras can promote the formation of a preorganized PIP2-binding-favored state in the catalytic subunit [52]. PIP3 is an important second messenger as it recruits several signaling proteins, including transducing kinases with pleckstrin homology domains, to the plasma membrane. One such serine/threonine kinase is AKT (alternatively, protein kinase B, PKB) and its activator PDK1 [53]. In the presence of increased PIP3 levels, PDK is localized near AKT and can phosphorylate the AKT molecule at the catalytic domain [54]. When mTOR complexes are also attracted to the PIP3-enriched membrane, mTORC2 can phosphorylate AKT in its hydrophobic domain [55]. Once AKT is activated and released from the membrane-bound protein complex, the downstream proliferative signals can be transmitted into the cytosol. AKT has more than 200 binding partners, ranging from the glucose intake regulator GSK3 to several cell cycle-controlling complexes to the p53 inhibitor MDM2; therefore, PI3K/AKT signaling has very diverse effects on cell growth and survival [53, 56]. Orchestration of spatial and temporal processes, such as complex signaling networks, requires fine-tuning of the players at every step of the signaling pathway. When Ras is mutated and the PI3K/AKT pathway is deregulated, multitudes of signaling molecules are affected, thus leading to uncontrolled cellular signaling [53].

It is not easy to investigate the involvement of Ras in the PI3K pathway because PI3K can also be activated by many other proteins independently from Ras, including G-protein-coupled receptors (GPCRs) and receptor tyrosine kinases (RTKs) [57]. However, the connection between Ras and PI3Kα seems to be essential, since blockage of their interaction in EGFR mutant-driven lung cancer can block tumor initiation and promote regression [58]. The PI3K pathway is frequently upregulated in RAS mutant cells, although Ras itself is insufficient to initiate PI3K-mediated tumorigenesis. During tumorigenesis, mutant Ras can interact with specific RTKs, and it has been shown that they can cooperatively activate PI3K in human colorectal [59] and lung cancer cell lines [60]. Inhibition of the PI3K pathway is insufficient to block malignant transformation because of a mutual connection between the PI3K and the MAPK pathways with a feedback mechanism, i.e., if one of the pathways is blocked, the second pathway is activated [61]. Therefore, to efficiently treat Ras-driven cancers, combination therapies may prove to be efficient.

We have already discussed how different amino acid substitutions have distinct consequences for MAPK pathway activation. The same is true for PI3K/AKT signaling, as mutations in Ras isoforms activate PI3K/AKT signaling with varying intensities. In an earlier work, Yan et al. studied the PI3K-activating potential of G12V mutant Ras isoforms, and they found that in transduced COS cells, H-Ras G12V could more robustly activate PI3K than could K-Ras G12V [62]. While activated Ras proteins trigger both the MAPK and PI3K/AKT pathways, the mechanisms underlying the regulation of the intensity of each pathway’s activation vary. For PI3K activation, the presence of GTP-bound Ras is sufficient. By contrast, Ras dimerization and, in the long term, Ras-formed microdomains are required for RAF and MAPK cascade initiation [63].

## Signaling via interactions between Ras and the guanine nucleotide exchange factors as TIAM1 or RalGDS

In addition to the two primary Ras-mediated pathways, Ras is also involved in several other signaling modalities. One of the less frequently studied pathways is RAC/PAK signaling. Activated Ras can interact with the Ras-binding domain of the TIAM1 protein (T lymphoma invasion and metastasis protein) at the plasma membrane [64]. TIAM1 is a guanine nucleotide exchange factor that facilitates the activation of RAC proteins. Via TIAM1 mediation, wild-type Ras can stimulate the GDP-GTP exchange of RAC, ultimately leading to the binding and phosphorylation of PAK serine threonine kinases (p21-activated kinases) [65]. PAK exerts its effects on many key regulatory proteins via phosphorylation, e.g., PI3K, RAF, and β-catenin, and it modulates cell growth and survival; however, it acts predominantly in cytoskeleton rearrangement and cell migration [66].

Mutant Ras can also upregulate RAC/PAK signaling. For example, several studies found elevated RAC activity in mutant HRAS-transformed fibroblasts [64, 67]. In a KRAS-driven skin squamous cell carcinoma mouse model, deletion of the PAK1 gene led to decreased tumor initiation and progression, thus indicating an important role for PAK1 in Ras signaling [68]. Additionally, mutant HRAS-driven PAKs play roles in cell cycle regulation by upregulating cyclin D1, a major activator of the G1-S transition, and this upregulation can cause malignant transformation [69]. PAKs are also involved in PI3K and RAF signaling, as they can activate certain components of these pathways. In many cases, therapeutic inhibition of the PI3K or RAF/MAPK pathways is ineffective [70, 71], perhaps in part due to PAK activation. Notably, PAK can also activate AKT, revealing that interplay exists between the major Ras pathways and RAC/PAK signaling.

It is plausible that combination therapies targeting members of these pathways and PAKs could work by preventing cross-activation; however, PAKs can act independently of the two abovementioned signaling pathways, since in mutant KRAS-driven colon cancer, knockdown of either PAK1 or PAK4 inhibits cancer cell proliferation and increases apoptosis [72]. Unfortunately, we currently only know a few effectors of RACs and PAKs, and the precise mechanisms by which they influence cellular processes are poorly understood. Given their significant roles in Ras-driven cancer, there is a great need for further investigation of these pathways.

There is an additional Ras effector pathway in which the initiation step of the signaling is triggered by an interaction between Ras and a guanine nucleotide exchange factor. RalGDS is a GEF for Ral and has also been identified as a Ras-binding partner. When Ras is in its active, GTP-bound state at the plasma membrane, RalGDS can bind to it to facilitate GDP to GTP exchange in Ral [73]. This mechanism is similar with that of the Ras/TIAM1/RAC activation complex, although Ras uses a non-Ras-specific GEF (e.g., TIAM1 and RalGDS) for the signal transduction. The Ras/RalGDS/Ral pathway was suggested to be involved in the regulation of anchorage-independent growth of Ras-driven colon cancer cells [74] and invadopodium formation of K-Ras mutant pancreatic adenocarcinoma [75].

## Death signaling modulated by Ras

Ras is mainly known because of its role in promoting cell survival and proliferation; however, in certain cases, mutant Ras can inhibit cell growth, and it can lead to cellular senescence or apoptosis [76, 77]. Matallanas et al. showed that mutant K-Ras can enhance apoptosis in a p53-dependent manner, and they suggested that wild-type K-Ras can counteract this proapoptotic effect [78]. Other groups have suggested a tumor suppressor role for wild-type K-Ras (rather than a transformation-promoting function) in different cancer types [79–81]. The context-dependent opposing effects of Ras are not a unique phenomenon among oncogenes, and these diverse processes are either oncogene-induced senescence or oncogene-induced apoptosis [82] [83–86].

Some key regulators of Ras-modulated cell death signaling pathways belong to the RASSF protein family [87]. The members of this family are scaffold proteins with no enzymatic activity, and RASSF1A and NORE1A are the two most studied RASSF family members [88, 89]. RASSF proteins have a Ras association (RA) domain by which Ras can directly activate them.

RASSF1A is one of the most frequently inactivated tumor suppressors in human cancer, presumably because it connects Ras signaling with apoptotic mechanisms [90, 91]. One of these apoptotic pathways is linked to Hippo protein kinase activation. RASSF1A can activate Hippo kinases, e.g., MST, leading to LAT kinase phosphorylation. At this step of the RASSF/Hippo pathway, the YAP and TAZ transcriptional regulators are recruited via phosphorylation, resulting in enhanced transcription of proapoptotic genes [92]. Elevated Ras activity can lead to cell death via apoptosis and prevent malignant transformation; thus, it is not surprising that Ras-driven tumors with RASSF1A inactivation have the poorest prognosis [87, 93].

NORE1A is the closest RASSF1A homolog, and while it is also a tumor suppressor, there are functional differences between the two proteins. While the main function of RASSF1A is to mediate Ras-driven apoptosis, it seems likely that NORE1A is primarily involved in cellular senescence (although it can also regulate apoptosis) [87]. Cellular senescence is a phenomenon that can prevent malignant transformation by arresting the cell cycle, as has been demonstrated for p53 and Rb tumor suppressors [94]. NORE1A can activate Rb via formation of a complex with PP1A, a phosphatase that can dephosphorylate Rb to activate it [95]. NORE1A also forms a complex with HIPK2, a kinase that can promote specific post-translational modifications of p53 that boost its senescence-promoting activity [96]. All of these NORE1A functions are regulated by Ras.

## Ras and phospholipase C interact in a signaling network

Phospholipase C (PLC) proteins are enzymes that can hydrolyze phosphatidylinositol 4,5-bisphosphate (PIP_2_) in the plasma membrane. This reaction produces two important intracellular second messengers, i.e., diacylglycerol (DAG) and inositol 1,4,5-trisphosphate (IP_3_), that lead to the initiation of several different downstream signaling pathways [97]. For instance, DAG mediates the activation of PKC, a major regulator of many cellular processes, including proliferation, oncogenic stress-induced apoptosis, and migration [98, 99]. Among the diverse functions of PKC, its cell motility enhancement activity is strongly linked to cancer development via its roles in controlling cell invasion and metastasis [100]. PKC has also been implicated in integrin-dependent signaling [101] and phorbol ester-induced cytoskeleton remodeling [102]. Furthermore, the PKCα isoenzyme is also linked to the regulation of intracellular trafficking by Ras, thereby modulating Ras-associated downstream signaling. Accordingly, PKCα can directly serine-phosphorylate the K-Ras4B hypervariable domain leading to Ras translocation from the cell membrane to the internal membranes. Ultimately, this phosphorylation event on K-Ras4b prevents signaling for survival and enhances proapoptotic mechanisms in certain cell types [103, 104].

The other PLC product IP_3_ is responsible for initiating Ca^2+^-dependent signaling in cells by enhancing Ca^2+^ release from intracellular stores [105]. Only one PLC protein, i.e., phospholipase C epsilon (PLCε), is linked directly to Ras [106]. PLCε has two Ras-associating domains (RA1 and RA2), and activated Ras can bind to the RA2 domain. This interaction markedly increases the enzymatic activity of PLCε [107]. Interestingly, PLCε is not only a Ras effector, it can also act as a Ras GEF via its CDC25 domain [108, 109]. Thus, the signal transduction downstream of the Ras-PLCε signaling node must be highly regulated; however, the precise mechanism of this regulation and the role of Ras in the Ca^2+^-dependent signaling network remain unclear. While it is clear that Ras can alter PLCε activity in some types of cancer [110], the role of PLCε in these disorders is unknown. For example, according to Bai et al., PLCε is an oncogene in H-Ras-triggered skin cancer [111]; however, a more recent investigation by another group suggested the opposite. Martins et al. concluded that PLCε may be a tumor suppressor in Ras-driven skin cancer based on their experiments in genetically engineered mouse models [112].

## Beyond the kinase cascades: the link between Ras signaling and miRNAs

Investigations of the frequent downregulation of microRNAs in various types of Ras mutant malignant cells led to the discovery that several miRNAs target the oncogenic Ras pathway. Since the primary functions of miRNAs are to limit translation or enhance the degradation of specific mRNAs, downregulation of Ras-targeting miRNAs could potentially upregulate Ras protein levels. For example, in colorectal cancers, in which Ras is a frequent oncogenic driving force, it was recently shown that the miR-143 level is decreased [113]. The potent anti-Ras activity of microRNAs can be exploited in tumor therapies. For example, Akao et al. showed that synthetic miR-143 could be used to silence K-Ras mRNA and that they could directly target AKT and ERK signaling in DLD-1 cells [114]. In breast cancer cells, miR-200c (also a Ras-targeting microRNA) affects various Ras pathway components thereby influencing AKT and ERK phosphorylation [115]. MicroRNAs can alter the Ras pathway by decreasing Ras mRNA translation; however, in some cases, a positive correlation between the expression levels of a microRNA and Ras has also been reported. It was recently observed that both the miR-21 and K-Ras expression levels were elevated in colorectal cancer samples, suggesting that, instead of suppressing the Ras pathway, miR-21 may promote K-Ras mRNA translation during cancer development [116]. Over the last decade, the pleiotropic effects and biological significance of microRNAs in Ras signaling modulation have been established, and the use of microRNA levels as biomarkers for cancer diagnosis is under extensive research [117, 118].

## Altered Ras signaling by mutant Ras forms in cancer stem cells

The cancer stem cell (CSC) concept proposes that tumor growth, like the renewal of healthy tissues, is fueled by a small subset of tumor cells endowed with stem cell characteristics. Over the past decades, most solid and non-solid tumors have been found to harbor CSCs or cells with stem cell (SC) features, such as self-renewal and quiescence (with exceptions) as well as expression of an often tumor-specific subset of SC markers. It has been demonstrated that CSCs contribute to tumor initiation, progression, metastasis, and therapy resistance and that this processes could be linked to aberrant Ras activation in CSCs [119, 120].

In colorectal cancer (CRC), a number of sequential genetic alterations drive tumorigenesis. Activating mutations in the KRAS gene alone do not usually induce transformation. Initiating genetic mutations, such as a loss-of-function mutation in the adenomatous polyposis coli (APC) gene, followed by accumulation of activating mutations in the KRAS gene are needed to drive tumorigenesis during the early and intermediate phases of CRC. The resulting aberrant activation of Wnt/β-catenin and Ras/ERK signaling are critical factors in the transformation and disease progression [121]. Loss of APC results in an initial activation of β-catenin signaling and K-Ras stabilization; subsequently, the activated β-catenin signaling is then further enhanced by stabilized mutant K-Ras, thus creating a positive feedback loop that promotes the development of CSC characteristics [121, 122]. Several studies have shown that mutations in APC and KRAS (e.g., G12D) can result in pronounced increases in both Wnt/β-catenin and Ras/ERK signaling activity, respectively, while CSC characteristics, such as sphere-forming capacity and the expression of CSC markers (i.e., CD44, CD133, and CD166), are increased [35, 121, 122].

The therapeutic response to gemcitabine (GEM), a standard chemotherapeutic agent used in the treatment of pancreatic adenocarcinomas, has proven to be unsatisfactory. Chemoresistance to GEM is associated with poor prognosis, and the reacquisition of CSC-like features is considered to be a main causative factor in the development of chemoresistance. Zhao et al. demonstrated that the mechanism behind GEM’s ineffectiveness was that after GEM induces NADPH oxidase activation via nuclear factor κB (NFκB), activated NADPH oxidase upregulates ROS production, which targets the K-Ras/MAPK pathway. In KRAS knockdown experiments, K-Ras was shown to be responsible for the GEM-mediated metabolic reprogramming and stemness of CSCs [123]. The K-Ras/JNK axis was shown to play a central role in maintaining CSCs or cancer stem-like cells (CSLCs) in pancreatic cancer [124]. As another example of how Ras is involved in CSC biology, Liu et al. [125] recently reported that urothelial carcinoma associated 1 (UCA1), a long noncoding RNA, is involved in the upregulation of K-Ras expression and activity in human pancreatic ductal adenocarcinoma cell lines. They showed that UCA1 can increase K-Ras expression at both the mRNA and protein levels by acting as a competing endogenous RNA sponging miR-590-3p, a suppressor of K-Ras expression. Moreover, upregulated UCA1 could increase the phospho-K-Ras level. Furthermore, sphere-formation assays revealed that UCA1 was also responsible for stemness maintenance by affecting the expression levels of the stem cell markers CD133, OCT4, Nanog, and SOX2 in human pancreatic ductal adenocarcinoma cell cultures [125], suggesting a UCA1/K-Ras/cancer stemness relationship. A recent study by Weng revealed additional Ras-mediated effects on CSCs and demonstrated that G12D mutant KRAS enhanced CSC marker expression (CD133, CD24, EpCAM) in prostate cancer [126]. As these and other studies demonstrate, Ras is a central player in CSC biology, and (depending on the type of tumor) several different molecules/pathways are involved in regulating its effects on CSC preservation and enrichment [127].

## Conclusions

Ras proteins coordinate multiple downstream effectors, many of which are aberrantly activated during cancer development. Although the regulatory roles of Ras proteins in signaling networks are mostly linked to the RAF/MEK/ERK kinase pathway and PI3K/AKT signaling, there is a growing evidence that Ras also mediates other signaling routes under normal and oncogenic conditions (Fig. 3). The four Ras isoforms differentially coordinate downstream effectors depending on their expression level or dimerization state. In addition to the various effects of Ras isoforms on signaling intensity, individual Ras mutants can strengthen or weaken signaling routes differently (Table 1).

It has been recently established that one of the ultimate challenges in anti-Ras therapy resides in Ras mutation-specific differences; Therefore, studies investigating the distinct roles of each Ras mutant in signal transduction should be prioritized to discover additional promising Ras mutation-selective therapeutic candidates.
